# Visualizing Alzheimer’s Disease Mouse Brain with Multispectral Optoacoustic Tomography using a Fluorescent probe, CDnir7

**DOI:** 10.1038/s41598-019-48329-4

**Published:** 2019-08-19

**Authors:** Sung-Jin Park, Chris Jun Hui Ho, Satoshi Arai, Animesh Samanta, Malini Olivo, Young-Tae Chang

**Affiliations:** 1 Singapore Bioimaging Consortium, Agency for Science, Technology and Research, Singapore, 138667 Republic of Singapore; 2 Research Institute for Science and Engineering, Waseda University, Tokyo, 169-8555 Japan; 3 PRIME, Japan Agency for Medical Research and Development (AMED), Tokyo, 100-0004 Japan; 4 Department of Chemistry, Pohang University of Science and Technology (POSTECH), Pohang, 37673 Republic of Korea; 5 Center for Self-assembly and Complexity, Institute for Basic Science (IBS), Pohang, 37673 Republic of Korea

**Keywords:** Small molecules, Photoacoustics

## Abstract

Alzheimer’s disease (AD) is now clinically considered as a chronic inflammation-based neurodegenerative disease. The CDnir7 probe was previously developed as an optical imaging probe to target macrophages in order to image mouse inflammation using *in vivo* optical imaging modalities such as *In Vivo* imaging system (IVIS) and fluorescent molecular tomography (FMT). Here, we demonstrate the application of CDnir7 in AD mouse brain imaging via multispectral optoacoustic tomography (MSOT). Longitudinal MSOT imaging of CDnir7 showed higher CDnir7 localization in AD mouse cerebral cortex compared to that of normal mice. MSOT signals of CDnir7 localization in mouse brain were verified by *ex vivo* near-infrared (NIR) imaging and immunohistochemistry. Histological evaluation showed strong CDnir7 staining in AD cerebral cortex, hippocampus, basal ganglia and thalamus area. Based on the supporting evidence, CDnir7 has great potential as a molecular imaging probe for AD brain imaging.

## Introduction

Alzheimer’s disease (AD) is a chronic inflammation in which microglia and macrophages accumulate in the cerebral cortex and hippocampus before visible symptoms such as amyloid-beta (Aβ) plaque formation, progress cognitive decline, irreversible memory loss, and emotional instability^1,2^. To detect AD, various molecular imaging techniques such as positron emission tomography (PET) have been developed, with Aβ plaque-targeting probes such as [^11^C]PIB^3,4^ and microglia/macrophages-targeting probes such as [^11^C]PK11195^5,6^. Although these molecular probes provided high molecular specificity, their spatial resolution is limited to 1–2 mm due to the positron range and the radiation dose, which hinders longitudinal inspection and reusability. For optical imaging, Aβ plaque-targeting fluorescent probes for *in vivo* imaging have been developed^7,8^. However, cell-specific targeting probes (e.g. macrophage targeting, astrocyte-targeting, etc) have not been well studied for penetration of the blood-brain barrier, depth of penetration, as well as sensitivity for brain imaging.

Multispectral optoacoustic tomography (MSOT) is an upcoming optical imaging modality that offers complementary advantages by combining high image contrast in optical imaging and high penetration depth in ultrasound imaging^9^. This MSOT technology has been studied for observing from intracellular organelles in cells to *in vivo* organ in small animals^10^, detecting cancer area^11^. In addition, such as upconversion nanopropbe^12^ and targeted near-infrared (NIR) fluorescent probes^13^ can be used in conjunction with MSOT imaging to increase the signal to background ratio in specific area of interest in the sample. Previous study has developed an optical imaging probe, CDnir7 (Compound of Designation near-infrared 7) that targets macrophages in an acute inflammation model induced by lipopolysaccharide or carrageenan injected into the paw region of mice, as well as the tumor region in an orthotropic mouse 4T1 breast cancer model. Accordingly, CDnir7 showed high localization in the inflamed regions – the inflamed paw and breast tumor region – via multiple imaging techniques, namely IVIS, FMT, and MSOT^13^. In this study, we demonstrate that, in the context of AD, this CDnir7 probe can also be used in MSOT imaging to distinguish AD mouse brains from healthy brains. Furthermore, we examined the CDnir7 staining pattern in AD mouse brain by processing sectioned brain samples using histological *ex vivo* methods such as NIR scanning and immunohistochemistry (IHC).

## Results

### MSOT showed CDnir7 stained AD mouse brain

**Figure 1:**
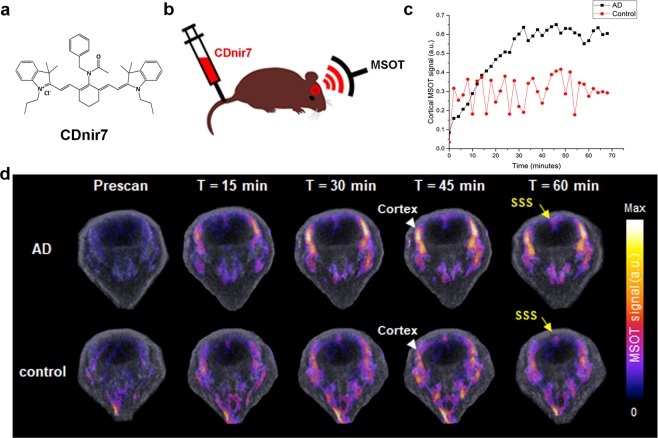
MSOT imaging of CDnir7 localization in the mouse brain. (**a**) Chemical structure of CDnir7. (**b**) Schematic of the experimental scheme for MSOT imaging of CDnir7 biodistribution in the mouse brain. (**c**) CDnir7 MSOT signals overlaid on OPUS anatomical images at various time points. (**d**) Longitudinal plot of CDnir7 MSOT signals in the cortex of both AD and control brains as a function of time. CDnir7 MSOT signals showed higher intensities in the cortex of AD brains compared to that of healthy controls from 20 minutes post-injection onwards, reaching a maximum AD-to-control difference at 30 minutes post-injection. In contrast, the CDnir7 MSOT signals at the superior sagittal sinus (SSS) of both AD and control brains showed similar intensities within the first hour post-injection.

CDnir7 was developed at excitation and emission wavelengths (λ_ex_/λ_em_ = 806/821 nm in DMSO) in the NIR range with low tissue autofluorescence. Although CDnir7 has a low fluorescence quantum yield (extinction coefficient = 198500 M^−1^ cm^−1^; quantum yield = 0.14) (Fig. 1a), it exhibits strong optoacoustic properties^13^. In this study, 13- and 15-month-old triple-transgenic AD mice (n = 5) were utilized for MSOT imaging since AD symptoms can be readily studied via histopathology from 12 months of age onwards. Before CDnir7 injection, the brain region from behind the orbital bone all the way up to the occipital bone in both AD and control mice was scanned *in vivo* via co-registered MSOT and ultrasound (OPUS), in order to obtain baseline images. Next, upon injection of 500 µM of CDnir7 via the tail vein, the same brain region in the mice was longitudinally imaged for an hour (Fig. 1b and Supplementary Movie 1). The resulting CDnir7 MSOT signals overlaid on OPUS anatomical images showed higher intensities in the cortex of AD brains compared to that of healthy controls (Fig. 1c,d) from 20 minutes post-injection onwards, reaching a maximum AD-to-control difference at 30 minutes post-injection. In contrast, the CDnir7 MSOT signals at the superior sagittal sinus (SSS) of both AD and control brains showed similar intensities (Fig. 1c) within the first-hour post-injection. Furthermore, when measuring total hemoglobin signals (oxygen-hemoglobin and dioxygen-hemoglobin) of both brains by MSOT, higher total hemoglobin signals were reversed in the cortex area of healthy control brain rather than that of AD brain (Supplementary Fig. 1). This demonstrates the high specificity of CDnir7 for the cortical region in AD brains, with similar CDnir7 concentrations in the blood circulation for both AD and control mice. These results showed that we can use CDnir7 in conjunction with MSOT imaging to clearly distinguish AD brains from healthy controls.

### Processed 3D (three-dimensional) MSOT images of CDnir7 signals in the mouse brain

**Figure 2:**
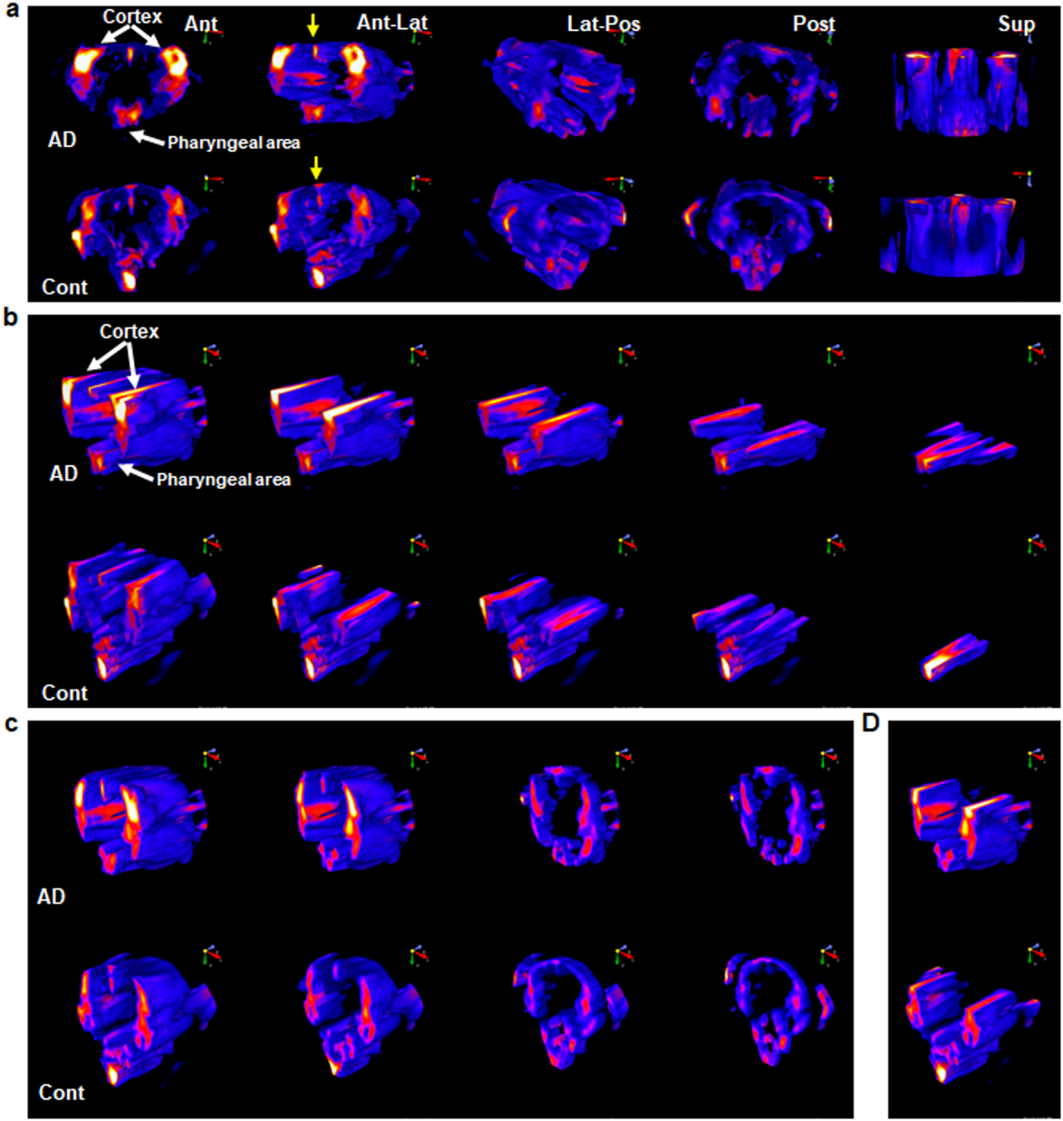
Processed 3D MSOT images of CDnir7 signals in the mouse brain. (**a**) 3D-rendered MSOT images of CDnir7 signals in the mouse brain in different orientations (Ant, anterior view; Ant-Lat, anterolateral view; Lat-Post, posterolateral view; Post, posterior view; Sup, superior view). CDnir7 MSOT signals showed higher intensities in the cortex of AD brains compared to that of healthy controls, but similar intensities at the superior sagittal sinus (SSS) (yellow arrows) in the brains of both AD and control mice. (**b**) MSOT images of brain CDnir7 signals sliced in different transverse planes. (**c**) MSOT images of brain CDnir7 signals sliced in different coronal planes. (**d**) MSOT images of brain CDnir7 signals sliced in both transverse and coronal planes.

For further image analysis, MSOT scanning data were exported from the MSOT system and input into NIS-Elements software to generate 3D-rendered MSOT images of CDnir7 signals in the brains of both AD and control mice. These 3D images were rotated in different orientations from anterior to posterior and with a superior view (Fig. 2a). Anterior and anterolateral (Ant-Lat) views of 3D-rendering images showed CDnir7 staining patterns in the cortex (white arrow), pharyngeal area and superior sagittal sinus (SSS, yellow arrows), but the CDnir7 signal intensity in AD disease brains was over 4-fold higher than that of healthy control brains (Fig. 2a). After this analysis, we digitally sliced the brains into different plane angles – transverse (Fig. 2b), coronal (Fig. 2c) and transverse-coronal (Fig. 2d) for in-depth image analysis. From these sliced analyses, it was confirmed that the cortex area in AD brains was significantly stained by CDnir7 (white arrows in Fig. 2b), but the SSS (yellow arrows) in the brains of both AD and control mice had similar intensities (Supplementary Movies 2 and 3). This suggested that CDnir7 circulation in the blood system is similar between AD and control brains, but AD brains have more CDnir7 retention properties in the cortex area. In contrast, the control mice had higher CDnir7 intensity signals in the pharyngeal area rather than in other brain regions, including the cortex. Figure 2b–d slice analyses confirmed our initial observations that CDnir7 MSOT signals showed higher intensities in the cortex of AD brains compared to that of healthy controls. From these results, we demonstrate the high specificity of CDnir7 for the cortical region of AD brains.

### Images of CDnir7-staining sectioned brain tissues

**Figure 3:**
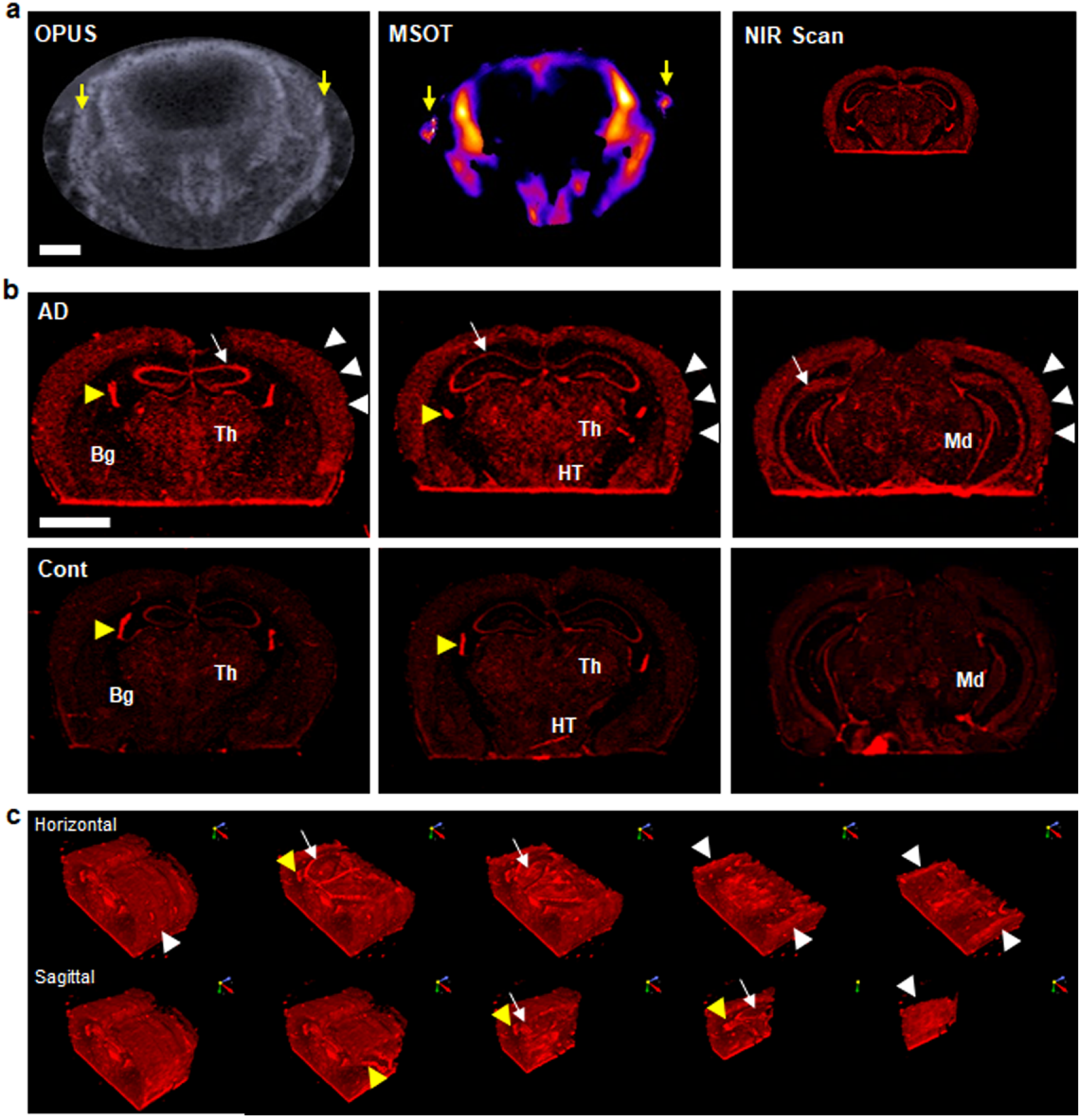
Images of CDnir7-stained sectioned brain tissues. (**a**) OPUS anatomical image of the mouse brain (left), CDnir7 MSOT image of CDnir7 localization (middle) and NIR fluorescence image of sectioned brain tissue showing CDnir7-stained patterns (right). Yellow arrow indicates the root of zygomatic bone. (**b**) NIR fluorescence image of sectioned brain tissue shows higher CDnir7 signal intensities in the hippocampus (white arrow) and cortex (white arrowheads) of the AD brain than those of the healthy brain. In contrast, similar CDnir7 signal intensities at the choroid plexus (yellow arrowhead) of both AD and healthy brains act as a positive control. (**c**) 3D-rendered NIR fluorescence images of sectioned brain tissue sliced in transverse and coronal planes showing CDnir7-stained patterns. Bg: basal ganglia, Th: thalamus, HT: hypothalamus, Md: midbrain. Scale bar, 2 mm.

Following MSOT imaging, the mouse brains were immediately enucleated, cryosectioned at 150 μm intervals, and then examined *ex vivo* for CDnir7 signal localization. Initially, the sectioned brain tissues were examined using the light-emitting diode (LED)-based fluorescence microscopy in the NIR range, but no CDnir7 signal of the sectioned brain tissues was observed (data not shown), which might be due to insufficient detection sensitivity of the microscopy system. Next, all sectioned brain tissues were scanned using a more sensitive NIR scanner (Odyssey-CLx LICOR^®^ with 21-µm resolution and high image quality options); representative images are shown in Supplementary Fig. 2. In order to compare *in vivo* MSOT/OPUS images with *ex vivo* NIR fluorescence images for examining CDnir7 signal localization in the mouse brain, both types of images need to be co-registered in the same spatial space. In this context, the positions of the hippocampus and the root of zygomatic bone were used as fiducial anatomical landmarks for image re-registration. Using our MRI scan database, the root of the zygomatic bone area was also matched with the hippocampal shape of the Fig. 3a NIR scan^14^. Figure 3a shows these aligned images between OPUS, MSOT and CDnir7 staining sectioned brain samples. Using this registration, we confirmed CDnir7 signal localization in the cortex. According to the hippocampal shapes in the brain, three representative CDnir7 NIR scan images were selected and are shown in Fig. 3b. CDnir7 signal intensities in the cortex (white arrowheads), hippocampus (white arrow), basal ganglia (Bg) and thalamus (Th) were much higher in the AD brains (Fig. 3b, upper row) than those in the healthy brains (Fig. 3b, lower row). In contrast, the CDnir7 signals in the choroid plexus (yellow arrowhead, Fig. 3b) of both AD and healthy brains were quite similar in intensity, which in turn acts as a positive control for the comparison. These staining patterns also strongly suggested that CDnir7 was injected in equal amounts into AD and healthy control brains, and that the highly staining cortex pattern of AD brains was generated by CDnir7.

For better 3D visualization of the CDnir7 stained patterns in these same AD brain regions in different orientations and planes (digitally horizontal and sagittal section images of 3D-rendered brains), the NIR images were co-registered to one another via MATLAB software and input into NIS-Elements software for further image processing (Fig. 3c and Supplementary Movie 4). These diverse digitally sectioned analyses of 3D-rendered images revealed CDnir7 highly stained the cortex (white arrowheads) and hippocampal areas (white arrows) in 3D-rendered structures of AD brains. The choroid plexus staining pattern for the CDnir7 injected positive control is also indicated with yellow arrowheads in these 3D-rendered structures. From these results, we demonstrate that the CDnir7 probe can be used to differentiate AD brains from healthy controls.

### Evaluation of CDnir7 cell-targeting ability via immunohistochemistry

**Figure 4:**
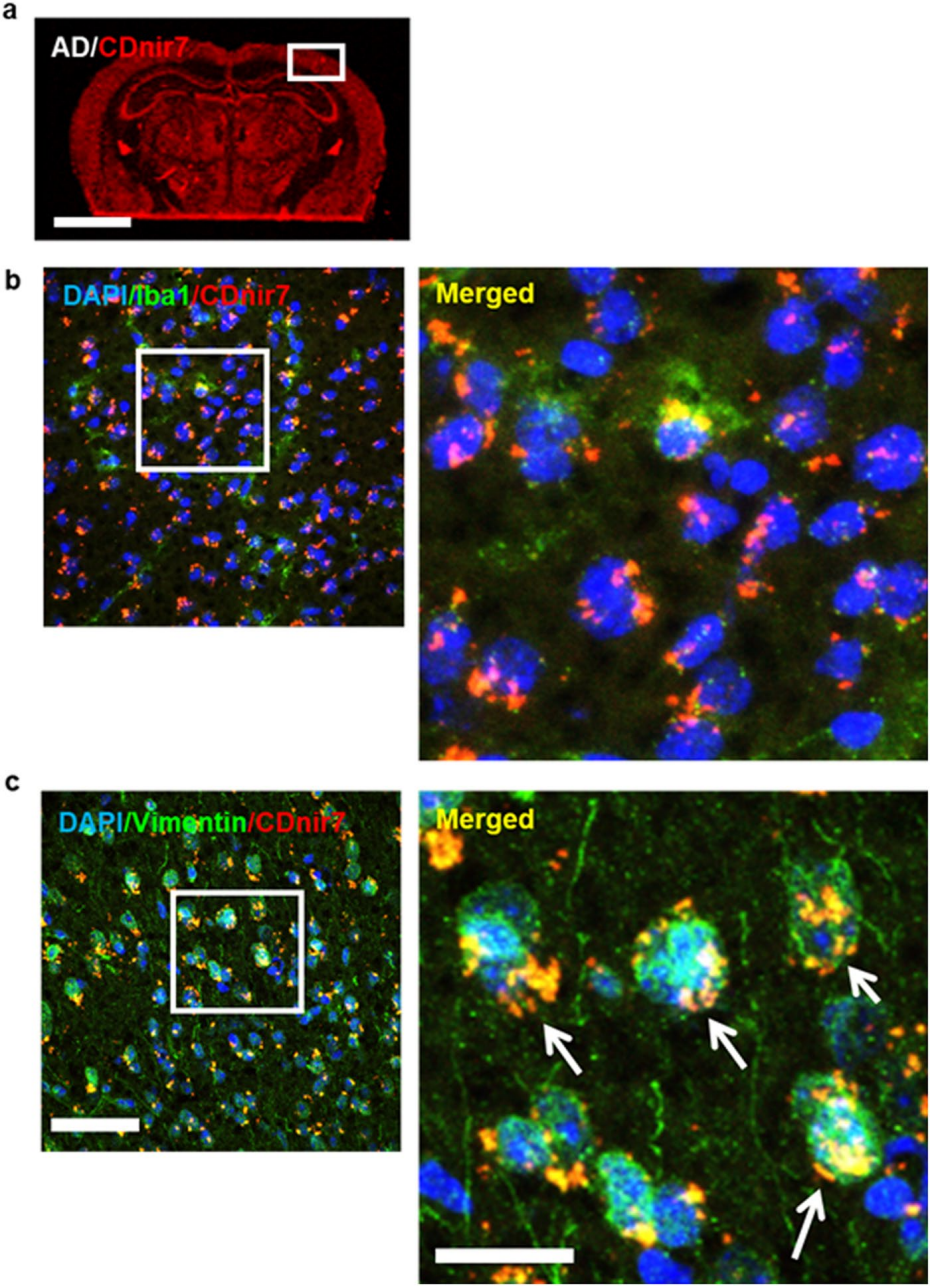
Evaluation of CDnir7 cell-targeting ability via immunohistochemistry. (**a**) NIR fluorescence image of sectioned AD brain tissue showing CDnir7 stained patterns. (**b**) Microglia/macrophage-specific anti-Iba1 stained patterns (green) overlaid on CDnir7 (red) signals showed low co-localization in the merged images. (**c**) Astrocyte-specific anti-vimentin stained patterns (green) overlaid on CDnir7 (red) signals showed high co-localization in the merged images. Scale bars, 50 *μ*m in the panel images and 20 *μ*m in the magnified images.

Next, the type of AD brain cells targeted by CDnir7 was determined via immunohistochemistry (IHC) experiments. Given that the fluorescence signals of CDnir7 were easily diminished after washing several times or a fixative step, the sectioned samples on the slides were first observed without any treatment by using a customized confocal microscope (Olympus) with a 633 nm laser excitation and a long-pass detection filter (BA650IF). After that, IHC was carried out and the same area initially observed was examined for the co-localization of CDnir7 and IHC positive cells. Since the previous studies^13^ demonstrated the macrophage-targeting ability of CDnir7 in both acute inflammation and 4T1 breast cancer models, the sectioned brain samples in this study were examined to confirm the microglia/macrophage-targeting ability of CDnir7 in the context of AD via immunohistochemistry (IHC), after being fixed with 4% paraformaldehyde. Surprisingly, CDnir7 staining patterns showed little to no co-localization with microglia/macrophages (anti-Iba1, Abcam, dilution factor, 1:100) in the AD brain cortex (Fig. 4b), but much higher co-localization with astrocytes instead (anti-vimentin, Abcam, dilution factor, 1:100), although staining patterns did not perfectly match due to morphological changes in the sample during IHC processing (Fig. 4c). The fluorescence of CDnir7 in the healthy brain cortex was lower than that in the AD brain cortex. However, as for the astrocyte staining pattern by the vimentin IHC, the difference was scarcely observed between the AD and healthy control brain cortex (Supplementary Fig. 3). In addition, we examined the intracellular localisation of CDnir7 with the co-staining of BODIPY^TM^ TR Ceramide (ThermoFisher Scientific, D7540), which is the fluorescent probe for detecting Golgi complex in live cells. For this experiment, we used the reactive SF268 astrocyte cancer cell line by ciliary neurotrophic factor (CNTF, 50 ng/ml, 4 days) treatment^15^. CDnir7 staining area was well co-localized in the Golgi complex (Supplementary Fig. 4). From these results, we demonstrate the astrocyte-targeting ability of CDnir7 in the context of AD and the Golgi complex stain of CDnir7 in the live reactive astrocyte cell line.

## Discussion

In this paper, we evaluated the application of an optical imaging probe, CDnir7, for differentiating AD brains from healthy controls via MSOT imaging and histopathology. From longitudinal MSOT imaging (Fig. 1), 3D MSOT image analysis (Fig. 2), histological *ex vivo* examination such as NIR fluorescence imaging (Fig. 3) and IHC (Fig. 4), we confirmed that CDnir7 successfully crosses the blood-brain barrier and targets astrocytes in the AD mouse brain cortex. To the best of our knowledge, CDnir7 is the first astrocyte-targeting small molecular probe for *in vivo* optoacoustic imaging in the AD mouse brain.

Surprisingly, the results from this CDnir7 brain imaging study were different from those of previous CDnir7-based studies in the acute inflammation and 4T1 mouse breast cancer models^13^, this may be due to pathological differences between these disease models. Acute inflammation causes a large number of immune cells to accumulate in the inflamed region and in the 4T1 breast cancer model at least half of the population of tumor cells displayed tumor-associated macrophages. In contrast, the AD mouse brain exhibits chronic inflammation with less macrophage accumulation. Furthermore, as mentioned above, CDnir7 can be easily washed away by PBS washing or fixation. These characteristics indicate that CDnir7 does not have any specific binding partner in cells and its targeting ability may be regulated via influx (endocytosis, SLC (solute carrier))^16^ or efflux transport systems (ABC, ATP-binding cassette transporter)^17^. ABC transporters have been well studied as some of them, such as ABC-B1, ABC-C1, and ABC-G2 transporters, contribute to drug resistance against cancer therapy. In order to study the relationship between CDnir7 and ABC transporters, we tested several ABC transporter inhibitors, such as KO143 (ABC-G2 inhibitor), Elacridar (ABC-B1/G2 inhibitor), MK574 (ABC-C1 inhibitor), Probenecid (ABC-B1/C3 inhibitor) and Verapamil (ABC-B1). Results showed that CDnir7 did not respond to any of these inhibitors in the Raw264.7 macrophage cell line (Supplementary Fig. 5). In addition, we also tested endocytosis inhibitors, such as Cytochalasin D for all types of endocytosis, LY294002 for micropinocytosis, nystatin for micropinocytosis, filipin III for clathrin-independent endocytosis, as well as phenylarsine oxide for phagocytosis. Once again, CDnir7 did not respond to any of these endocytosis inhibitors (Supplementary Fig. 6). Taken together, these results suggest that CDnir7 may be more involved in the SLC system, rather than the ABC transporter or endocytosis systems. The SLC system has more than 400 SLC gene subtypes and a quarter of SLC genes are associated with various human diseases^16^, but many gene families are still relatively unexplored. The intracellular localization test showed that the stained Golgi complex by BODIPY^TM^ TR ceramide was co-localized by CDnir7. It suggested that Golgi complex related SLC system such as SLC35 family of nucleotide sugar transporters and SCL50 sugar transporter^16,18^ may be involved in CDnir7 stain pattern in astrocytes (Supplementary Fig. 4).

Furthermore, during AD progression, Aβ accumulation induced reactive astrocytes, which produced inflammatory chemokines^19,20^ and complement^21^, and reduced glutamate uptake^22^. In light of these properties of reactive astrocytes, metabolic change within a transporter system can induce the uptake of CDnir7 in AD brains. The results from this study suggest some physiological differences between AD and healthy astrocytes in mouse brain, which may be related to the SLC system. Thus, future SLC expression studies in astrocytes may help to provide a better understanding of the astrocyte-targeting mechanism of CDnir7. Such insights into drug resistance or transporter-related mechanisms, in turn, can lead to the future development of drugs and imaging probes. This study demonstrates that an astrocyte targeting mechanism of CDnir7 has not yet been elucidated, but CDnir7 has great potential as a molecular imaging probe in the AD mouse brains.

## Methods

### Animals and MSOT scanning

All animal experimental procedures were performed in accordance with a protocol approved by the Institutional Animal Care and Use Committee for Biological Resource Center at A*STAR, Singapore (IACUC #161108 and #171284). 13- to 15-month-old triple transgenic female AD (B6;129-*Psen1*^*tm1Mpm*^ Tg(APPSwe,tauP301L)1Lfa/Mmjax, n = 5) and control (C57/BL6, n = 5) mice were utilized for MSOT imaging, since AD symptoms can be readily studied via histopathology from 12 months of age onwards. 500 µM CDnir7 with 5% DMSO and 0.1% Tween-20 in PBS was injected via the tail vein for MSOT imaging. MSOT images were acquired using the MSOT inVision 512-echo system (iThera Medical GmbH, Munich, Germany). The excitation source is a tunable optical parametric oscillator laser with a wavelength range from 660 to 1300 nm, providing light excitation on the sample via a fiber bundle. The detector is a transducer array with 512 elements, at a central frequency of 5 MHz. This transducer array is in the shape of a circular arc spanning 270 degrees, for detection of optoacoustic signals within the arc at high sensitivity. Both the fiber bundle output and the transducer array are immersed in a water bath maintained at 34 degrees Celsius. During optoacoustic data acquisition, the sample is translated through the transducer array along its axis and cross-sectional image slices of the region-of-interest are acquired, with an inter-slice gap of 0.5 mm. Detected optoacoustic raw signals are averaged over 10 consecutive laser pulses for each wavelength (715, 730, 760, 800 and 850 nm) and axial position.

The raw MSOT signals were processed prior to image formation via the back-projection algorithm in the proprietary ViewMSOT software suite (ver3.8; iThera Medical). After reconstruction, the optoacoustic images acquired at different wavelengths were linearly unmixed on a per-pixel basis to differentiate the spectral signatures of tissue chromophores (deoxy-hemoglobin, Hb; oxy-hemoglobin, HbO_2_; dye, CDnir7). The tissue chromophores were resolved spatially and quantitatively, and then overlaid in a pseudo-colour scheme to form an image representing spatial distribution of the different bio-chromophores in the tissue.

### NIR scanning observation and three-dimensional rendering for whole sectioned brain samples

After MSOT imaging, the mice were euthanized, and the mouse brains were enucleated and cryo-sectioned in preparation for *ex vivo* histological analysis. In particular, the mouse brain region from the rostral to the caudal hippocampus was sectioned using cryostat equipment (Leica, CM1950) with a slice thickness of 12 µm and inter-slice gap of 150 µm. The sectioned brain samples were attached to slides, which in turn were imaged using a NIR scanner (Odyssey-CLx LICOR^®^) with 21 µm resolution and high image quality options.

MSOT and NIR fluorescence images were exported from the MSOT system and the NIR scanner respectively and input into NIS-Elements software for generating corresponding 3D-rendered images and movie files, rotated in different orientations and digitally sliced in different planes.

### Immunohistochemistry observation for CDnir7 positive cells

CDnir7 stained patterns in the sectioned brain samples were imaged using the FV1000 confocal microscope (Olympus) with an objective lens (UPlanFL, 20X, NA  N0.50). They were fixed with 4% paraformaldehyde and washed out with PBS. Then, the samples were treated with 1% bovine serum albumin for 1 hour to eliminate non-specific binding. The antibodies used were astrocyte-specific anti-vimentin (dilution factor, 1:100 Abcam) and microglia/macrophage-specific anti-Iba1 (dilution factor, 1:100, Abcam). For the chromogen, the Alexa Fluor^TM^ 594 conjugated secondary antibodies were used for IHC, in order to reduce the interference between CDnir7 signals and IHC signals. Subsequently, after Hoechst staining, the processed brain samples were stained with DAPI (λ_ex_/λ_em_ = 405/430–450 nm) and Alexa Fluor^TM^ 594(λ_ex_/λ_em_ = 559/575–620 nm) and then imaged. CDnir7 was excited with a 635 nm laser and its fluorescence emission was recorded, using a filter (BA655–755). Although the fluorescence signal of CDnir7 was declined after fixation process, the weak but effective signal could be observed in the far-red region of fluorescence.

### Intracellular localization of CDnir7

For examining the intracellular localization of CDnir7, SF295 cell line which is astrocyte cancer cell line was used and was induced to reactive astrocytes by ciliary neurotrophic factor (CNTF, 50 ng/mL) for 4 days^15^. After that, we examined the co-localization of CDnir7 and Golgi complex staining fluorescent probe, which was BODIPY^TM^ TR ceramide (ThermoFisher Scientific, D7540, λ_ex_/λ_em_ = λ589/617 nm). For reconstructing the BODIPY^TM^ TR ceramide fluorescent probe, we followed the company protocol. Briefly, we prepared sphingolipid-BSA (bovine serum albumin) complex and did the reconstitution of ready-made ceramide-BSA complexes. For staining, the cells were rinsed by 25 mM HEPES buffer and incubated with 30 min at 4 °C with 5 uM ceramide-BSA complexes in HEPES. After washing with HEPES buffer at 4 °C, the cells were incubated in fresh medium and CDnir7 (1 uM) at 37 °C for 30 min. The staining cells were observed by the customized Nikon Eclipse Ti microscopy.

## Supplementary information

Supplementary Information

Supplementary Movie 1

Supplementary Movie 2

Supplementary Movie 3

Supplementary Movie 4

## Data Availability

Any supplementary information and videos are available in the online version of the paper. All other data are available from the authors upon request.
