# Supplementary material for: Visualizing Alzheimer’s Disease Mouse Brain with Multispectral Optoacoustic Tomography using a Fluorescent probe, CDnir7
Source: Sci Rep. 2019 Aug 19;9:12052. doi: 10.1038/s41598-019-48329-4 (PMC6700105; doi:10.1038/s41598-019-48329-4)
Supplement: Supplementary file 1 — Supplementary Information [file 41598_2019_48329_MOESM1_ESM.pdf]

Supplementary Information for

Visualizing Alzheimer's Disease Mouse Brain with Multispectral Optoacoustic Tomography using a Fluorescent Probe, CDnir7

Park, *et al.*

Supplementary Information for

Visualizing Alzheimer's Disease Mouse Brain with Multispectral Optoacoustic Tomography using a Fluorescent probe, CDnir7

Sung-Jin Park[$], Chris Jun Hui Ho[$], Satoshi Arai[$], Animesh Samanta, Malini Olivo, Young-Tae Chang*

[$]These authors contributed equally to this work.

*Correspondence to Young-Tae Chang (ytchang@postech.ac.kr)

Contents of this SI file include:

- Supplementary Figure 1 to 6

Other Supplementary information for this manuscript include:

- Supplementary Movie 1 to 4.

List of Supplementary Figures:

1. **Supplementary Figure 1.** Total haemoglobin signals in control and AD brains by MSOT.
2. **Supplementary Figure 2.** CDnir7 stained pattern in serial sectioned AD brain
3. **Supplementary Figure 3.** Comparison of CDnir7 and vimentin IHC fluorescence images of sectioned AD and healthy control brain tissues.
4. **Supplementary Figure 4.** Intracellular localization of CDnir7
5. **Supplementary Figure 5.** CDnir7 stained pattern in ABC transporter inhibitor treatment.
6. **Supplementary Figure 6.** CDnir7 stained pattern in endocytosis inhibitor treatment.

List of Supplementary Movies:

7. **Supplementary Movie 1.** CDnir7 time trace by MSOT.
8. **Supplementary Movie 2.** CDnir7 stain pattern in 3D reconstruction of control brain.
9. **Supplementary Movie 3.** CDnir7 stain pattern in 3D reconstruction of AD brain.
10. **Supplementary Movie 4.** 3D reconstruction of CDnir7 stained AD brain sections.

Supplementary Results

Supplementary Figure 1

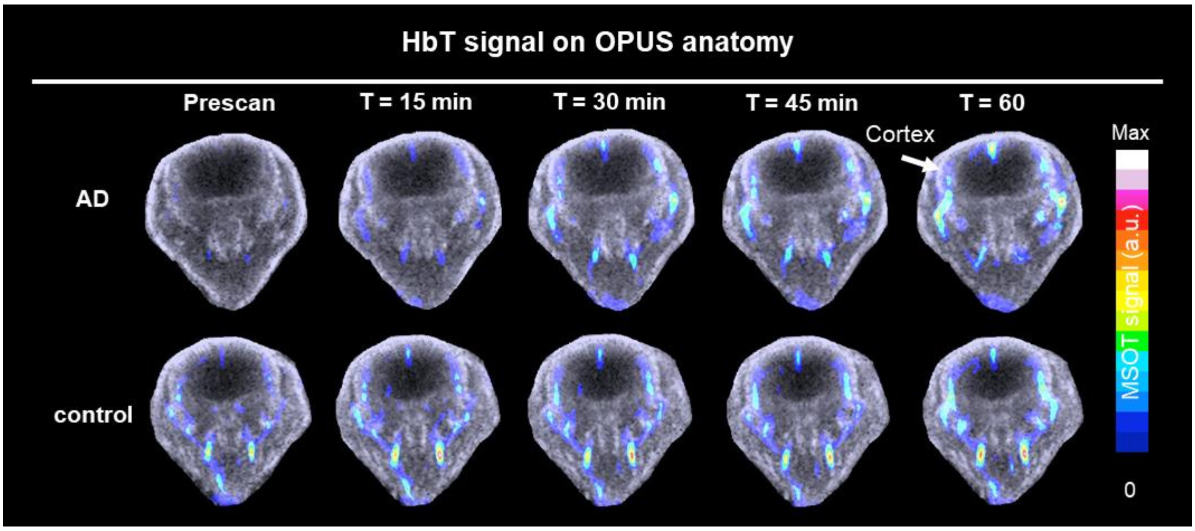

Supplementary Figure 1. Total haemoglobin signals in control and AD brains by MSOT. The total haemoglobin (HbT; oxy- and deoxy-haemoglobin) signal analysis by MSOT showed there were no high signals in cortex area in AD brain during 1hr observation.

Supplementary Figure 2

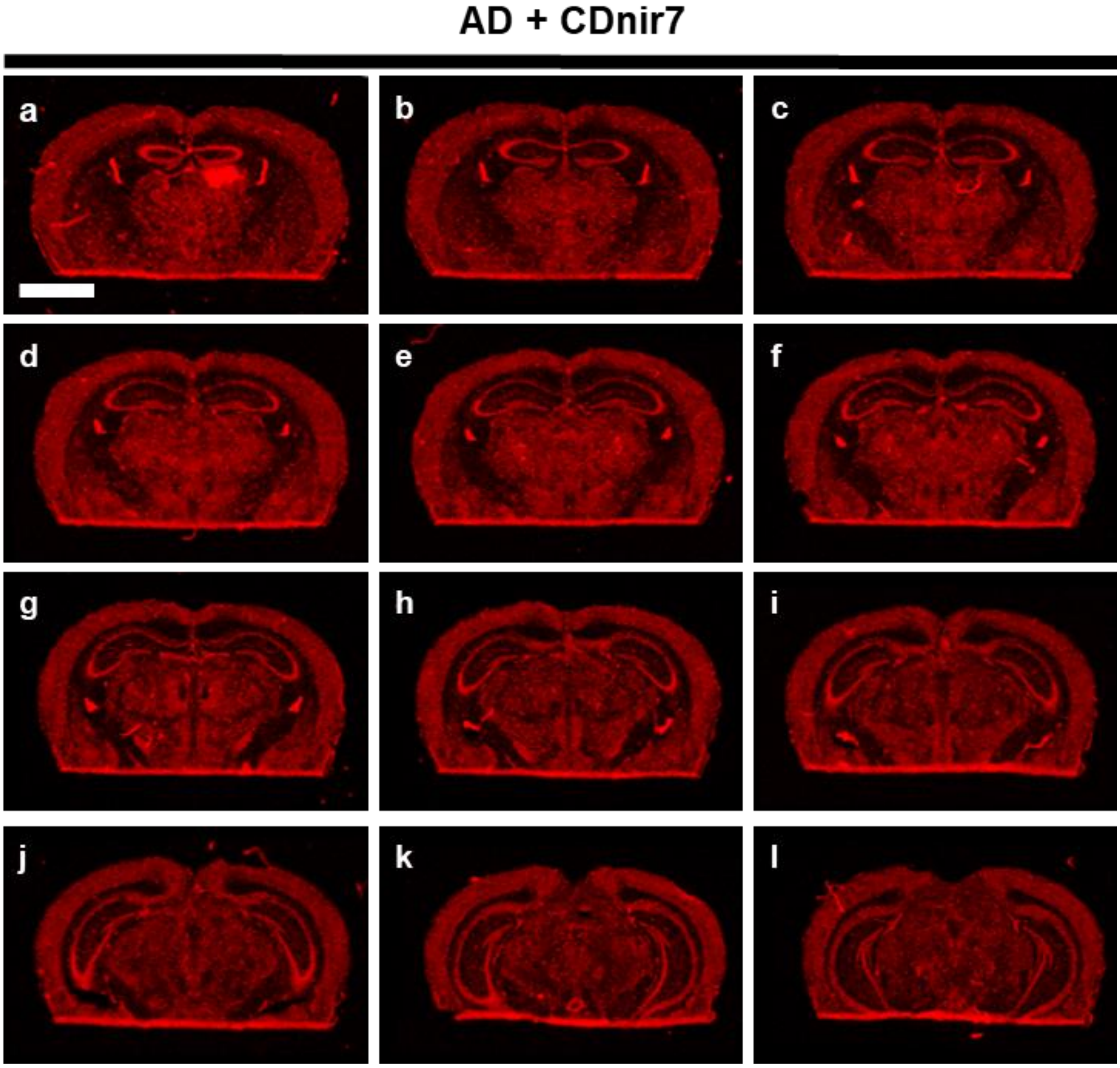

Supplementary Figure 2. CDnir7 stained pattern in serial sectioned AD brain. a-l. CDnir7 stained brain section images in hippocampal located area of AD brain. The interval of the sectioned brain images was 300μm. Scale bar, 2mm.

Supplementary Figure 3

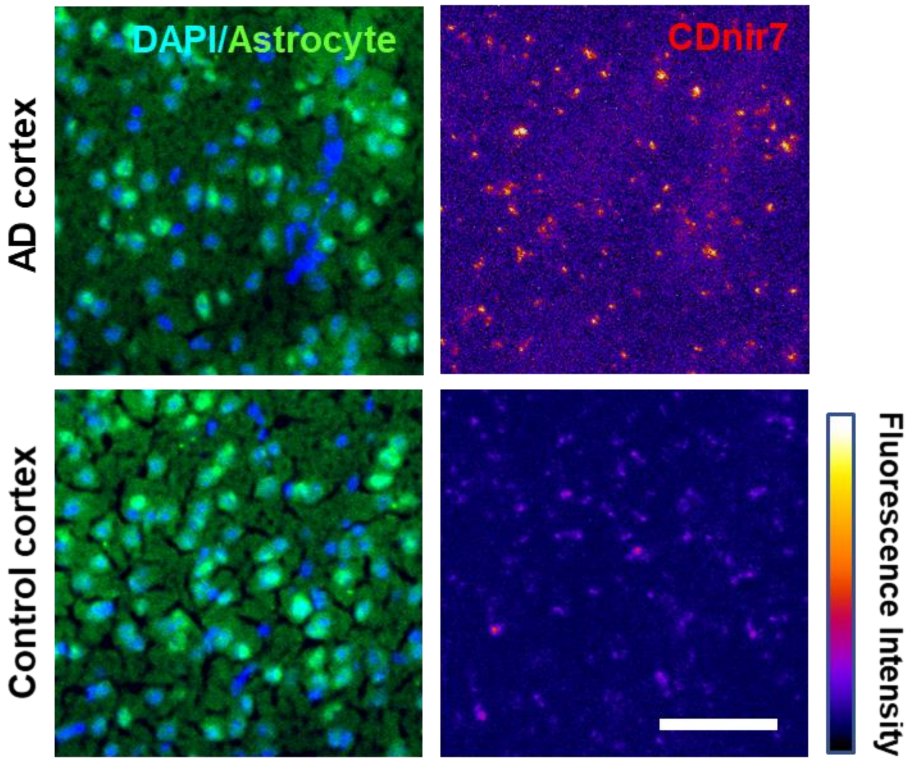

Supplementary Figure 3. Comparison of CDnir7 and vimentin IHC fluorescence images of sectioned AD and healthy control brain tissues. CDnir7 and vimentin IHC fluorescence images of AD and control healthy brain cortex tissues were taken by the confocal microscopy. Left panel: DAPI (Blue) and Astrocyte (Green, vimentin IHC) staining. Right panel: CDnir7 fluorescent images after tail vein injection. The sectioned images of CDnir7 were shown at the same pseudo colour range. The scale bar, 50 μm.

Supplementary Figure 4

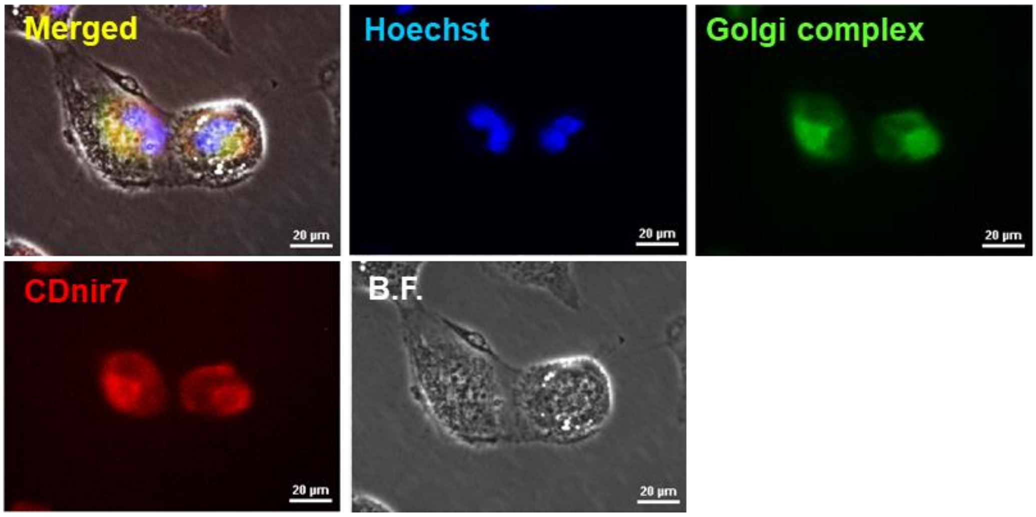

Supplementary Figure 4. Intracellular localization of CDnir7. Reactive SF268 astrocyte cancer cell line by CNTF was stained by CDnir7 and BODIPY^{TM} TR ceramide, which stains the Golgi complex. CDnir7 staining intracellular area was co-localized with the Golgi complex. Scale bar, 20μm

Supplementary Figure 5

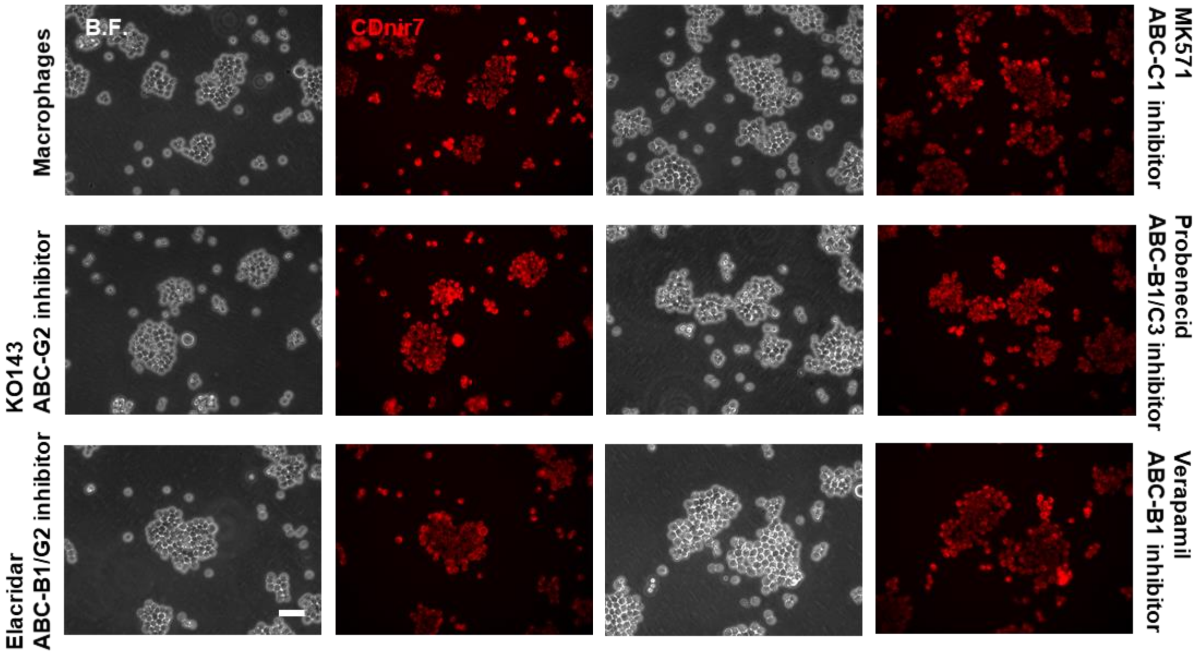

Supplementary Figure 5. CDnir7 stained pattern in ABC transporter inhibitor treatment. CDnir7 stained patterns in Raw246.7 cell lines were examined under ABC transporter inhibitors such as KO143 (ABC-G2 inhibition), Elacridar (ABC-B1/G2 inhibition), MK571 (ABC-C1 inhibition), Probenecid (ABC-B1/C3 inhibition) and Verapamil (ABC-B1 inhibition), but there were no prominent responses against each inhibitor. Scale bar, 100μm.

Supplementary Figure 6

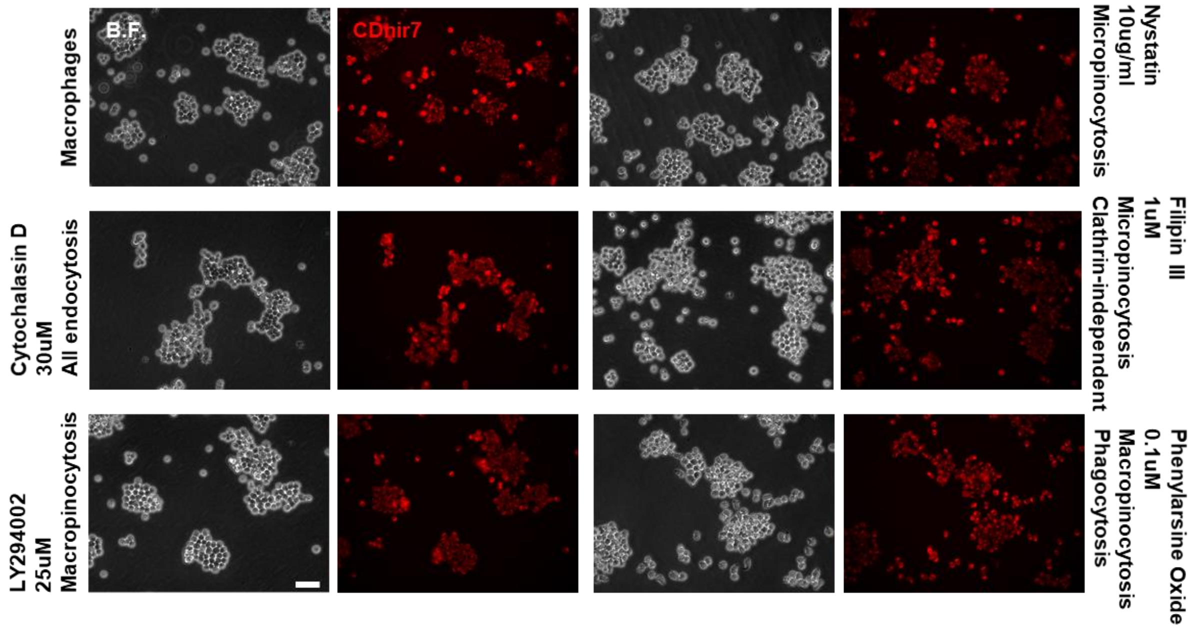

Supplementary Figure 6. CDnir7 stained pattern in endocytosis inhibitor treatment. CDnir7 stained patterns in Raw246.7 cell lines were examined under endocytosis inhibitors, as well. Cytochalasin D for all endocytosis inhibition, LY294002 for acropinocytosis inhibition, nystatin for micropinocytosis inhibition, filipin III for microphinocytosis and clathrin-independent inhibition, phenylarsine oxide for micropinocytosis and phagocytosis inhibition were utilised but there were no prominent different between the control and the inhibitor treated groups. Scale bar, 100μm.
